# Proteomic screening of plasma identifies potential noninvasive biomarkers associated with significant/advanced fibrosis in patients with nonalcoholic fatty liver disease

**DOI:** 10.1042/BSR20190395

**Published:** 2020-01-03

**Authors:** Wei Hou, Michael G. Janech, Philip M. Sobolesky, Alison M. Bland, Salma Samsuddin, William Alazawi, Wing-Kin Syn

**Affiliations:** 1 Division of Gastroenterology and Hepatology, Department of Medicine, Medical University of South Carolina, Charleston, SC, U.S.A.; 2 Tianjin Second People’s Hospital and Tianjin Institute of Hepatology, Tianjin, China; 3 Department of Medicine, Nephrology Proteomics Laboratory, Medical University of South Carolina, Charleston, SC, U.S.A.; 4 Hollings Marine Laboratory, Department of Biology, College of Charleston, Charleston, SC, U.S.A.; 5 Department of Pathology, Center for Advanced Laboratory Medicine, University of California San Diego, San Diego, CA, U.S.A.; 6 Blizard Institute, Queen Mary, University of London, London, U.K.; 7 Section of Gastroenterology, Ralph H. Johnson Veterans Affairs Medical Center, Charleston, SC, U.S.A.; 8 Department of Physiology, Faculty of Medicine and Nursing, University of the Basque Country (UPV/EHU), Leioa, Spain

**Keywords:** Complement, Liver fibrosis, NAFLD, Noninvasive biomarker, Proteomics

## Abstract

Noninvasive biomarkers are clinically useful for evaluating liver fibrosis stage in patients with nonalcoholic fatty liver disease (NAFLD). The aim of the present study was to compare plasma proteins in patients with early nonalcoholic steatohepatitis (NASH) (F0-F1) versus NASH with significant/advanced fibrosis (F2–F4) to determine whether candidate proteins could be used as potential noninvasive biomarkers. Nineteen biopsy-proven NAFLD patients including ten early NASH patients and nine NASH patients with significant/advanced fibrosis were enrolled in the present study. High-resolution proteomics screening of plasma was performed with the SCIEX TripleTOF 5600 System. Proteins were quantified using two different software platforms, Progenesis Qi and Scaffold Q+, respectively. Progenesis Qi analysis resulted in the discovery of 277 proteins compared with 235 proteins in Scaffold Q+. Five consensus proteins (i.e. Complement component C7; α-2-macroglobulin; Complement component C8 γ chain; Fibulin-1; α-1-antichymotrypsin) were identified. Complement component C7 was three-fold higher in the NASH group with significant/advanced fibrosis (F2–F4) compared with the early NASH (F0-F1) group (q-value = 3.6E-6). Complement component C7 and Fibulin-1 are positively correlated with liver stiffness (*P*=0.000, *P*=0.002, respectively); whereas, Complement component C8 γ chain is negatively correlated (*P*=0.009). High levels of Complement C7 are associated with NASH with significant/advanced fibrosis and Complement C7 is a perfect classifier of patients included in this pilot study. Further studies will be needed in a larger validation cohort to confirm the utility of complement proteins as biomarkers or mechanistic determinants of NASH with significant/advanced fibrosis.

## Introduction

Nonalcoholic fatty liver disease (NAFLD), one of the most common liver diseases worldwide, can be categorized histologically into nonalcoholic fatty liver (NAFL) or nonalcoholic steatohepatitis (NASH) [1]. Accurately evaluating liver fibrosis stage in NAFLD patients is of importance for identifying those who may progress to liver cirrhosis and hepatocellular carcinoma. Although a liver biopsy is the gold standard for diagnosing and determining the stages of fibrosis, there is an urgent need to develop noninvasive methods [2–12], including imaging modalities [13–17], biomarkers [18–26], and artificial intelligence algorithms [27–32].

The purpose of the present study was to perform proteomic screening of plasma via SCIEX TripleTOF 5600 System [33–35] to identify potential noninvasive biomarkers in NAFLD patients with significant/advanced liver fibrosis.

## Methods

### Patients

Plasma samples were acquired through the ‘Prevalence and Risk Factors for NAFLD in patients with T2DM’ study conducted at the Royal London Hospital, Barts Health NHS Trust, United Kingdom (U.K.). The study was approved by the National Research Ethic Service (Reference 14/WA/1142) and carried out in accordance with the World Medical Association Declaration of Helsinki. Written informed consent was obtained from all participants.

### Histological evaluation

All patients enrolled in the present study had undergone a percutaneous liver biopsy. Fibrosis stage was scored according to the Kleiner classification [36] on a 5-point scale (F0–F4).

### Clinical and biochemical data

Relevant clinical data were recorded, including the patients’ age, sex, weight, and height. Body mass index (BMI) was calculated as weight (kg) divided by height (m) squared. Venous blood samples were collected in lithium-heparin tubes and plasma was stored at −80°C until analysis. Laboratory evaluation in all patients included measurement of the serum levels of aspartate aminotransferase (AST) and alanine aminotransferase (ALT). Liver stiffness for all patients were assessed by FibroScan.

### Proteomics and data processing

Plasma was assayed for total protein (Bio-Rad Protein Assay) and 5 µl was digested with trypsin (1:10 ratio enzyme to protein) for 18 h at 37°C following following reduction and alkylation as we previously described [37]. Peptides were desalted using solid phase extraction cartridges (Strata-x, Phenomenex) and eluted sequentially in 25% acetonitrile/0.1% formic acid and 45% acetonitrile/0.1% formic acid. Each peptide fraction was diluted 1:4000 in 0.1% formic acid and 10 µl injected on to a 2-cm c18 trap column (Dionex pepmap100, Thermo Scientific). Peptides were separated on a c18 analytical column (15 cm × 75 µm, Dionex pepmap 100) for 60 min from 0.1% formic acid to 50% acetonitrile/0.1% formic acid. Data were acquired in IDA mode on a SCIEX 5600. Wiff files were uploaded to Progenesis Qi or converted into .MGFs for MASCOT searching (v2.4) against the human proteome database (SwissProt/UniProt; 2015) before being loaded to Scaffold Q+. Both Progenesis Qi and Scaffold Q+ were utilized to determine significant protein differences to construct a consensus differential protein list to reduce analytical bias of a single software platform. Proteomics data of the present study has been loaded into the ProteomeXchange database (Submission Reference No.: 1-20190126-112361).

### Statistical analysis

Statistical differences in Progenesis Qi were determined using ANOVA of log2 transformed data and *P*-values corrected for local false discovery rate (q-value). Proteins with *q*-values less than 0.05 were considered different. Statistical differences in Scaffold Q+ were determined using a *t* test of normalized spectral abundance factors (NSAF) as a comparison with Progenesis Qi results. NSAF values were not transformed for normality, nor were *P*-values adjusted for multiple comparisons. For statistically different proteins (Progenesis Qi), receiver operating characteristic (ROC) curves were created in Sigmaplot plotted using the data from Progenesis Qi, and the areas under the ROC curves (AUROCs) were calculated in order to represent classification performance. Correlation analysis was performed by using the Pearson’s correlation coefficient test in SPSS (IBM). Differences were considered to be statistically significant at *P*<0.05.

## Results

### Characteristics of NAFLD patients

A total of 19 biopsy-proven NAFLD patients were enrolled in the present study, including 10 early NASH patients and 9 NASH patients with significant/advanced fibrosis. The characteristics of the patients are summarized in Table 1. There were no significant differences in terms of sex, age, BMI, ALT, AST and smoking between the two groups. Patients with significant/advanced NASH fibrosis had elevated liver stiffness (*P*<0.001), Fibrosis-4 (FIB-4) score (*P*<0.01), and a higher prevalence of diabetes (*P*<0.001).

**Table 1:** Clinical characteristics of the patients enrolled in the study

	Early NASH (F0-1)	Advanced NASH (F2–4)	*P*-value
**Sex (M/F)**	(4/6)	(5/4)	0.65
**Age (Mean [Range], Years)**	55.6 [35–70]	58.4 [47–74]	0.56
**BMI**	31 ± 4	32 ± 4	0.38
**Diabetes (No. Pts)**	1	9	<0.001
**Stiffness (kPa)**	5.8 ±1.7	19.7 ± 9.8	<0.001
**FIB4**	1.1 ± 0.2	2.1 ±0.9	<0.01
**ALT**	44 ± 34	65 ± 29	0.34
**AST**	34 ± 20	51 ± 19	0.14
**Smoker (%)**	90%	89%	1

### Differentially abundant plasma proteins

**Figure 1:**
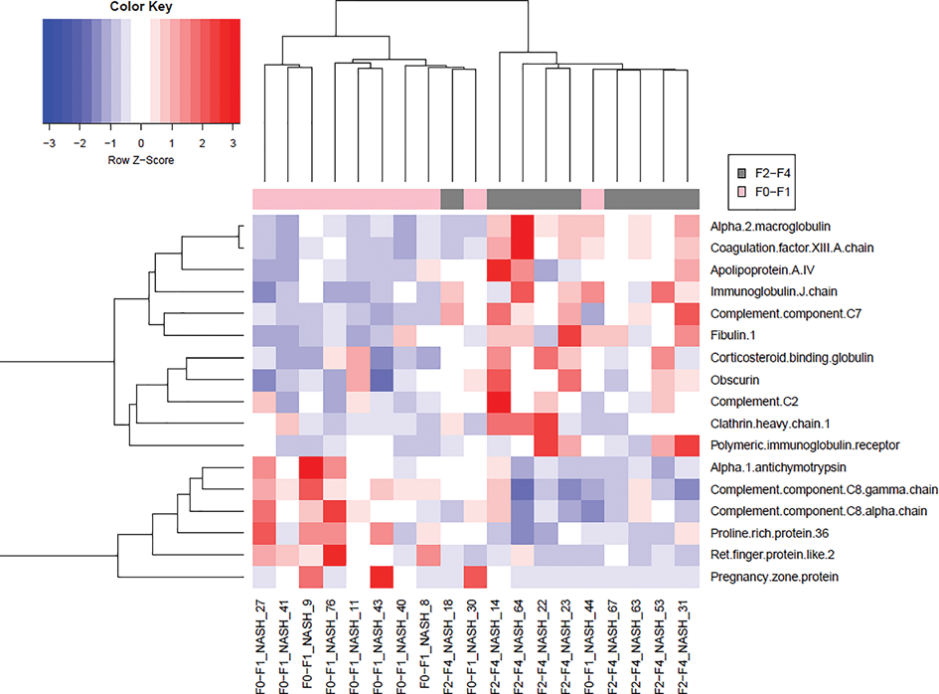
Hierarchal clustering heat map of significant plasma proteins as discovered by Progenesis Qi analysis Hierarchal clustering of 17 significantly different proteins was utilized to group patients using an average (UPGMA) agglomeration method in R Studio. Color intensity indicates standard deviations from the mean (Z-score) in a positive (red) or negative (blue) direction.

**Figure 2:**
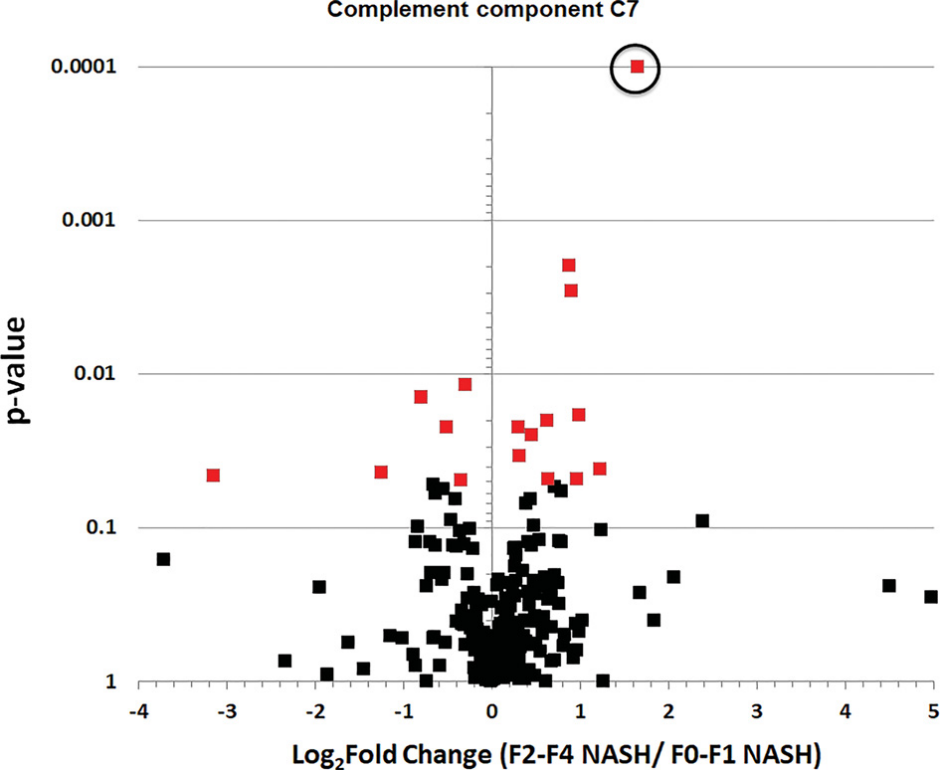
The volcano plot of all proteins identified in the Progenesis Qi analysis Complement C7 was circled above and was three-fold higher (log2 = 1.65) in the NASH group with significant/advanced fibrosis (F2–F4) compared with the early NASH (F0-F1) group (actual q-value = 3.6E-6, but was changed to 0.0001 for display purposes).

Progenesis Qi workflow analysis resulted in the discovery of 277 proteins compared with 235 proteins in Scaffold Q+ workflow. Five consensus proteins (i.e. Complement component C7; α-2-macroglobulin; Complement component C8 γ chain; Fibulin-1; α-1-antichymotrypsin) were shown in Table 2. Hierarchal clustering of 17 significantly different proteins from the Progenesis Qi workflow was utilized to group patients using an average (UPGMA) agglomeration method in R Studio. Color intensity indicates standard deviations from the mean (Z-score) in a positive (red) or negative (blue) direction (Figure 1). The volcano plot (log2 fold change vs. *P*-value) displays all proteins identified in the Progenesis Qi analysis (Figure 2). Complement component C7 was three-fold higher (log2 = 1.65) in the NASH group (F2–F4) with significant/advanced fibrosis compared with the early NASH (F0-F1) group (q-value = 3.6E-6).

**Table 2:** Identification of five consensus proteins via Progenesis Qi and Scaffold Q+ analysis in NASH patients with significant/advanced fibrosis versus early NASH patients

Protein name	Progenesis Qi (total proteins = 277)	Scaffold Q (total proteins = 235)
Complement component C7	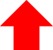	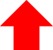
α-2-macroglobulin	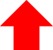	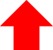
Complement component C8 γ chain	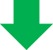	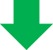
Fibulin-1 OS = *Homo sapiens*	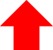	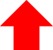
α-1-antichymotrypsin	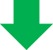	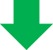

### ROC curves

**Figure 3:**
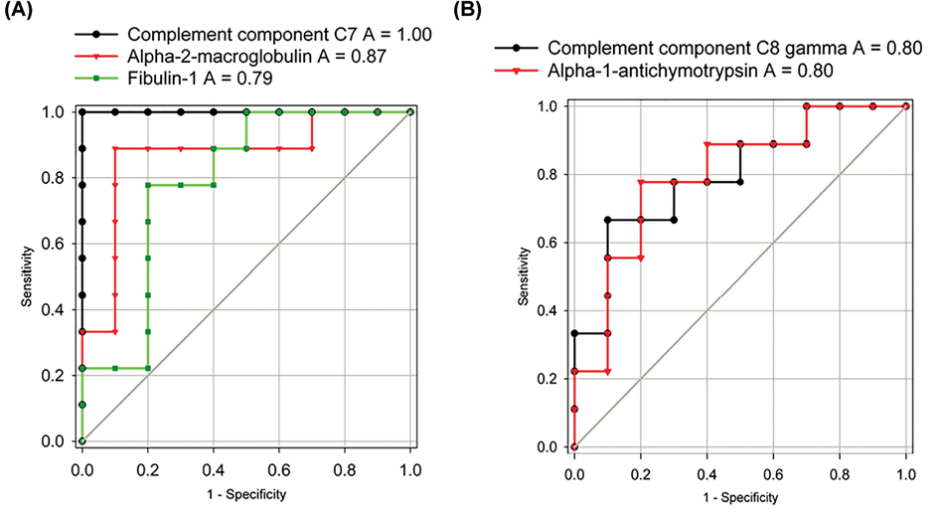
Classification performance estimated using ROC curve for the five consensus differential plasma proteins (**A**) Proteins elevated in the NASH patients with significant/advanced fibrosis (F2–F4). (**B**) Proteins lower in the NASH patients with significant/advanced fibrosis (F2–F4). Abbreviation: AUC, area under the curve. All areas have a *P*-value <0.03.

Classification performance was estimated using AUROCs for the five consensus different proteins identified. As shown in Figure 3A, the AUROCs for those proteins elevated in NASH patients with significant/advanced fibrosis (F2–F4) are 1.00 (Complement component C7), 0.87 (α-2-macroglobulin), and 0.79 (Fibulin-1), respectively. As shown in Figure 3B, the AUROCs for those proteins reduced in NASH patients with significant/advanced fibrosis (F2–F4) are both 0.80 (Complement component C8 γ chain; α-1-antichymotrypsin).

### Correlation analysis

As shown in Table 3, Complement component C7 and Fibulin-1 are positively correlated with liver stiffness (*P*=0.000, *P*=0.002, respectively); whereas, Complement component C8 γ chain is negatively correlated (*P*=0.009). Moreover, Complement C7 and α-2-macroglobulin were positively correlated with FIB-4 (*P*=0.031, *P*=0.042, respectively) while Complement component C8 γ chain was negatively correlated with FIB-4 (*P*=0.013). Interestingly, Complement component C7 also positively correlated with HBA1c (*P*=0.045) while γ-1-antichymotrypsin is negatively correlated with ALT (*P*=0.036).

**Table 3:** Pearson’s r correlation analysis of five consensus differential plasma proteins

		Stiffness	Complement C7	α-2- macroglobulin	Complement C8 γ chain	Fibulin-1	α-1-antichymotrypsin
**Stiffness**	Pearson correlation	-	**0.740****^†^**	0.407	−**0.584****^†^**	**0.657****^†^**	−0.453
	Sig. (two-tailed)	-	**0.000**	0.084	**0.009**	**0.002**	0.051
	*n*	-	**19**	19	**19**	**19**	19
**FIB-4**	Pearson correlation	0.502	0.556*	0.530*	−0.623*	0.297	−0.330
	Sig. (two-tailed)	0.056	**0.031**	**0.042**	**0.013**	0.282	0.230
	*n*	15	**15**	15	**15**	15	15
**ALT**	Pearson correlation	0.126	0.037	0.185	−0.459	0.320	−0.511*
	Sig. (two-tailed)	0.629	0.887	0.477	0.064	0.210	**0.036**
	*n*	17	17	17	17	17	**17**
**HBA1c**	Pearson correlation	0.539	0.613*	0.438	−0.077	0.010	0.068
	Sig. (two-tailed)	0.087	**0.045**	0.178	0.821	0.977	0.843
	*n*	11	**11**	11	11	11	11
**BMI**	Pearson Correlation	0.277	0.228	0.264	−0.167	0.267	0.072
	Sig. (two-tailed)	0.251	0.347	0.274	0.494	0.270	0.770
	N	19	19	19	19	19	19

* Correlation is significant at the 0.05 level (two-tailed).

† Correlation is significant at the 0.01 level (two-tailed).

## Discussion

The present study showed for the first time that Complement C7 and C8 γ chain and Fibulin-1 significantly correlate with liver stiffness. Moreover, Complement C7 acted as a potential biomarker to identify those NASH patients with significant/advanced liver fibrosis.

The complement system, a phylogenetically ancient makeup of the humoral system, consisting of a cascade of proteases and soluble factors, plays a vital role in innate immune [38]. The role of complement in liver diseases is less well characterized. The complement components C3 and C5 were reported to be associated with NAFLD [39–41] and C5 had a causal role in liver fibrogenesis [42]. There are strong and persistent stimuli for complement activation in NAFLD through multiple pathways, including the classical pathway and the lectin pathway [43], as well as the alternative pathway [44]. The complement component 7 (C7) is a terminal component of the complement cascade. The role C7 plays in the pathogenesis and progression of NAFLD are largely unknown. Our results showed that C7 was three-fold higher in the NASH group with significant/advanced fibrosis (F2–F4) compared with the early NASH (F0-F1) group, consistent with previous studies [45], suggesting C7 might be an important contributor in the pathogenesis of NAFLD. The exact mechanisms of C7 in the progress of NAFLD need to be further elucidated.

One limitation of our pilot study is the relatively small number of patients. Nevertheless, the sample numbers were sufficient to show significant differences in plasma proteins between early NASH and NASH with significant/advanced fibrosis in a conceptual approach.

In summary, the present study demonstrated that Complement C7 and C8 γ chain as well as Fibulin-1 significantly correlated with liver stiffness. Elevated levels of Complement C7 were detected in NASH patients with significant/advanced fibrosis and were a perfect classifier for patients included in this pilot study. Further studies in a larger validation cohort will be needed to confirm the utility of complement proteins as potential biomarkers or mechanistic determinants of progressive NASH.

## Perspectives

- Accurately evaluating liver fibrosis stage in NAFLD is important because it enables the identification of those most likely to progress to liver cirrhosis and hepatocellular carcinoma. Although a liver biopsy remains the current gold standard for diagnosis and staging of liver disease, it is invasive, costly, and with inherent risks; hence there is an urgent need to develop noninvasive methods.
- Proteomic screening of plasma via SCIEX TripleTOF 5600 System identified potential noninvasive biomarkers of NAFLD patients with significant/advanced liver fibrosis. Complement components C7 and C8 γ chain, and Fibulin-1 significantly correlated with liver stiffness.
- In this pilot, high levels of Complement C7 were associated with NASH with significant/advanced fibrosis and Complement C7 is a perfect classifier of patients with NASH.

## Abbreviations

ALT: alanine aminotransferase
AST: aspartate aminotransferase
AUROC: area under the ROC curve
BMI: body mass index
FIB-4: Fibrosis-4
NAFLD: nonalcoholic fatty liver disease
NASH: nonalcoholic steatohepatitis
NSAF: normalized spectral abundance factor
ROC: receiver operating characteristic
